# Intratumoral and peritumoral MRI habitat imaging for differentiating stage IA endometrial cancer from benign endometrial lesions: a multicenter study

**DOI:** 10.3389/fonc.2026.1790637

**Published:** 2026-07-09

**Authors:** Yunzhu Wu, Xianhong Wang, Cheng Deng, Qiu Bi, Yang Song, Shenghong Ju, Yang Liu, Conghui Ai, Jing Yang

**Affiliations:** 1 Jiangsu Key Laboratory of Intelligent Medical Image Computing, School of Artificial Intelligence, Nanjing University of Information Science and Technology, Nanjing, China; 2 Chuxiong Medical College, Chuxiong, Yunnan, China; 3 Department of Radiology, The Second Affiliated Hospital of Kunming Medical University, Kunming, Yunnan, China; 4 Department of MRI, The First People’s Hospital of Yunnan Province, The Affiliated Hospital of Kunming University of Science and Technology, Kunming, Yunnan, China; 5 MR Research Collaboration Team, Siemens Healthineers Ltd., Nanjing, China; 6 Department of Radiology, Zhongda Hospital, School of Medicine, Southeast University, Nanjing, China; 7 Department of Radiology, The First Affiliated Hospital of Chongqing Medical University, Chongqing, China; 8 Department of Radiology, Yunnan Cancer Hospital, The Third Affiliated Hospital of Kunming Medical University, Peking University Cancer Hospital Yunnan, Kunming, Yunnan, China

**Keywords:** benign endometrial lesions, endometrial carcinoma, habitat imaging, magnetic resonance imaging, peritumoral microenvironment

## Abstract

**Purpose:**

The aim of this study was to evaluate the value of different multiparametric MRI-based radiomics models in differentiating stage IA endometrial cancer (EC) from benign endometrial lesions.

**Methods:**

This retrospective study included 787 patients with stage IA endometrial cancer (EC) or benign endometrial lesions from four centers. Tumor regions of interest (ROIs) were manually delineated on MRI. Employing Python, the following peritumoral ROIs were automatically generated: 3-mm dilated and eroded peritumoral loops (LDE3), 3-mm eroded peritumoral loops (LE3), and intratumoral regions merged with 3-mm dilated peritumoral loops (RD3) Habitat clustering was performed using K-means and Gaussian Mixture Model (GMM) algorithms. Logistic regression was utilized to identify independent predictors and construct habitat-only and combined (clinical + habitat) models. Performance was evaluated using the area under the curve (AUC).

**Results:**

Age (P = 0.002) and vaginal bleeding (*P* < 0.001) were identified as independent clinical predictors of stage IA EC. Habitat-based models outperformed the clinical model in some external validation cohorts. Peritumoral features, particularly K-means_RD3, exhibited favorable robustness, achieving an average external validation AUC of 0.740. The integration of habitat features with clinical predictors yielded synergistic improvements, with the Clinical+K-means_RD3 model achieving the highest diagnostic performance (peak AUC: 0.921). Notably, this combined framework effectively mitigated the limitations of clinical factors in challenging subsets, elevating the AUC from 0.644 to 0.850 in validation group D. K-means demonstrated superior robustness and stability compared to GMM.

**Conclusion:**

Intratumoral and peritumoral habitat imaging based on multiparametric MRI can non-invasively reveal the microstructural characteristics of stage IA EC. Integrating clinical predictors with habitat models showed good diagnostic performance. The peritumoral habitat model exceeded the intratumoral habitat model.

## 1 Introduction

Endometrial carcinoma (EC) is a prevalent gynecological cancer that affects women globally, with an increasing incidence in recent years (1). International Federation of Gynecology and Obstetrics (FIGO) stage IA is defined as tumors confined to the endometrium or with a myometrial invasion depth of less than 50% (2), which often overlaps with benign endometrial lesions, such as endometrial hyperplasia or polyps, in clinical and imaging features (3). This brings challenges to preoperative differential diagnosis, and the accuracy of its diagnosis directly affects the choice of treatment strategies, including whether to preserve fertility (4). Although traditional diagnostic modalities, including endometrial cytology, endometrial biopsy and dilation and curettement, are regarded as the gold standard, they are invasive, are associated with sampling errors, and not applicable to all patient populations (5, 6). Therefore, the development of a non-invasive and accurate imaging approach holds crucial significance for guiding clinical decision-making and mitigating both overtreatment and undertreatment.

Although MRI is the standard imaging modality for staging endometrial cancer, its specificity in differentiating stage IA EC from benign lesions remains limited, with a reported diagnostic accuracy of 68.2% (7). Conventional MRI relies on visual assessment of signal intensity and morphology, which is subjective and fails to capture the sub-visual intratumoral heterogeneity and peritumoral microenvironmental changes. Habitat imaging, an AI method that utilizes unsupervised clustering algorithms, categorizes tumors into distinct subregions to delineate the spatial dissection of tumor heterogeneity (8). At present, functional habitats based on contrast-enhanced T1-weighted and DWI sequences has demonstrated clinical value in predicting the IDH status of glioma, among other applications (9). In addition to intratumoral heterogeneity, peritumoral regions warrant attention. The peritumoral region, or the tumor-myometrial interface, is an active site for tumor angiogenesis and can be utilized to predict lymph node metastasis (LNM) and lymph vascular space invasion (LVSI) status of EC patients (10). Our previous research shows that both intra- and peri-tumoral MRI radiomics can be used to noninvasively predict deep myometrial invasion of early-stage endometrioid adenocarcinoma (11). Furthermore, a study has demonstrated the potential of habitat analysis based on GMM and K-means clustering in distinguishing uterine sarcoma from atypical leiomyoma, finding that the performance of GMM was superior to that of K-means (12).Therefore, we speculate that characterizing the habitats of the microenvironment surrounding the tumor could provide valuable and sensitive diagnostic markers for differentiating stage IA EC from benign endometrial lesions. To date, most non-invasive diagnostic models have focused solely on intratumoral radiomics features.

Our study is the first to leverage both intratumoral and peritumoral MRI-based habitat imaging using unsupervised clustering techniques (GMM and K-means) in a large multicenter cohort to distinguish stage IA EC from benign lesions.

## 2 Materials and methods

### 2.1 Study population

**Figure 1:**
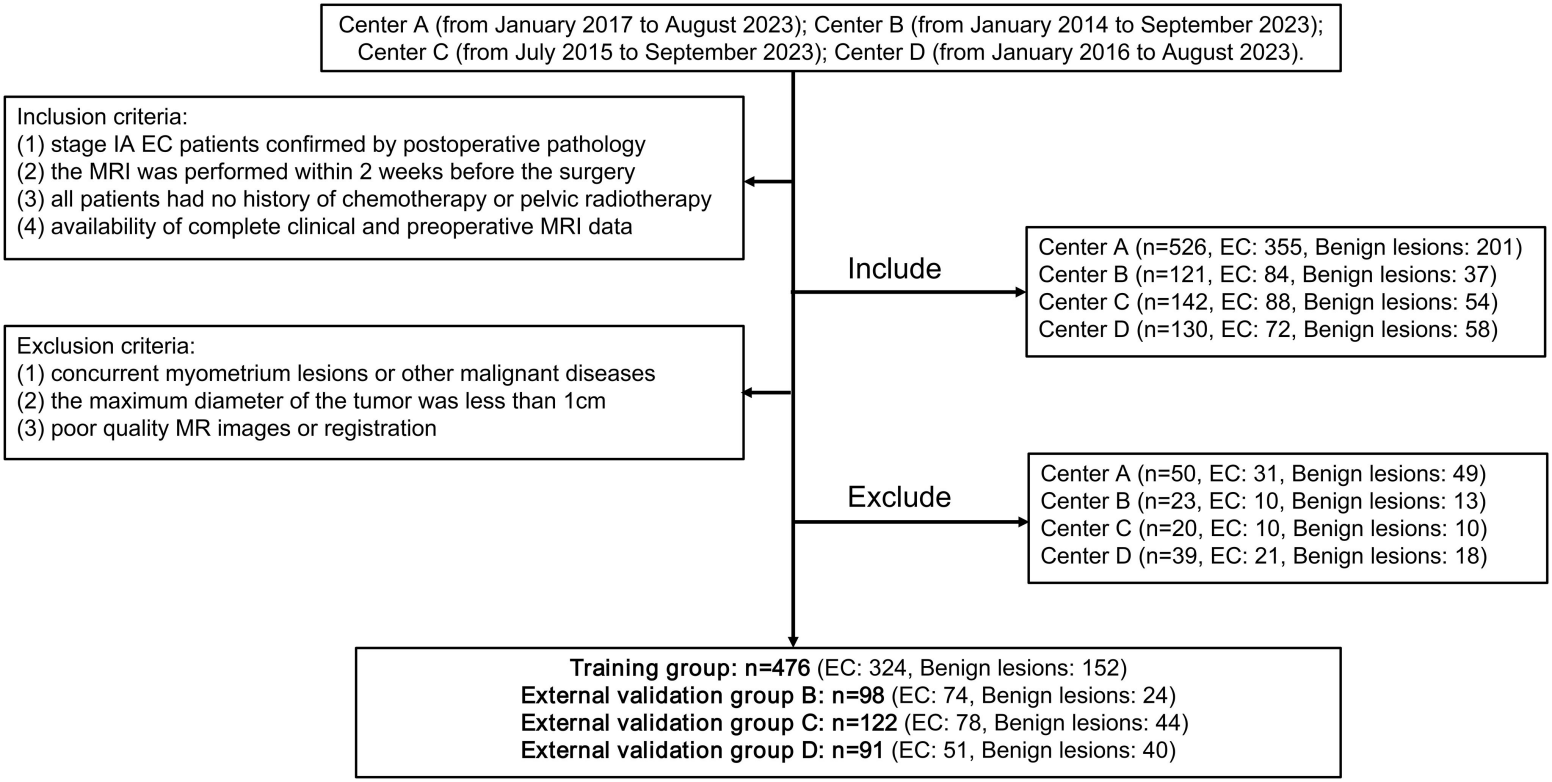
Flowchart of the patient recruitment and exclusion. EC, endometrial cancer.

The ethics committees of the four centers approved this retrospective study, and informed consent was waived. This study collected data from patients diagnosed with stage IA EC between 2014 and 2023. Patients from Center A (The First People’s Hospital of Yunnan Province, Kunming, China) were included in the training cohort, while those from Centers B (The Second Affiliated Hospital of Kunming Medical University, Kunming, China), C (Peking University Cancer Hospital Yunnan, Kunming, China), and D (The First Affiliated Hospital of Chongqing Medical University, Chongqing, China)were included in the validation cohort based on the following inclusion criteria: (1) patients with anatomical stage IA EC according to the 2023 FIGO staging system, confirmed by postoperative pathology; (2) preoperative pelvic MRI was performed within 2 weeks before the surgery; (3) all patients had no history of chemotherapy or pelvic radiotherapy; and (4) availability of complete clinical and preoperative MRI data. MRI sequences used included axial T2-weighted imaging (T2WI), apparent diffusion coefficient (ADC) maps, and contrast-enhanced T1-weighted imaging (CE-T1WI). The exclusion criteria were as follows: (1) concurrent myometrial lesions or other malignant diseases; (2) maximum tumor diameter of less than 1cm; or (3) poor quality MR images or registration. The flowchart of patient enrollment is shown in **Figure 1**.

The clinical data collected included: (1) general clinical information including: age, body mass index, myometrial invasion, tumor grade, histological subtype, irregular vaginal bleeding, menopausal status, hypertension, and diabetes; (2) laboratory tests including: CA125 and CA199.

### 2.2 MRI protocol and processing

All MR examinations were performed using 1.5T/3.0T scanners (Siemens Prisma, Siemens Aera, Siemens Skyra, GE Signa HDX, GE Premier, GE Pioneer, and Philips Ingenia) with patients in the supine position. The imaging sequences included axial T2WI, DWI (b-value = 1000 s/mm^2^), and CE-T1WI. CE-T1WI images were obtained approximately 3 minutes after intravenous gadolinium administration (0.2 ml/kg), at a rate of 2 ml/s. More detailed sequence scanning parameters are presented in **Supplementary Table 1**. Axial T2WI, ADC maps, and CE-T1WI images in digital imaging and communications in medicine (DICOM) format were exported from the picture archiving and communication system for analysis. The images were preprocessed using 3D Slicer software (version 4.11.0, https://www.slicer.org/) and the PyRadiomics library (version 3.1.0) implemented in Python (version 3.11.4; https://www.python.org/). The resample image (BRAINS) module adjusted the voxel size to 1 × 1 × 1 mm^3^, followed by spatial alignment using the general registration (BRAINS) module. Additionally, N4 bias field correction was implemented to address magnetic field non-uniformity across different scanners.

### 2.3 Habitat segmentation and clustering

**Figure 2:**
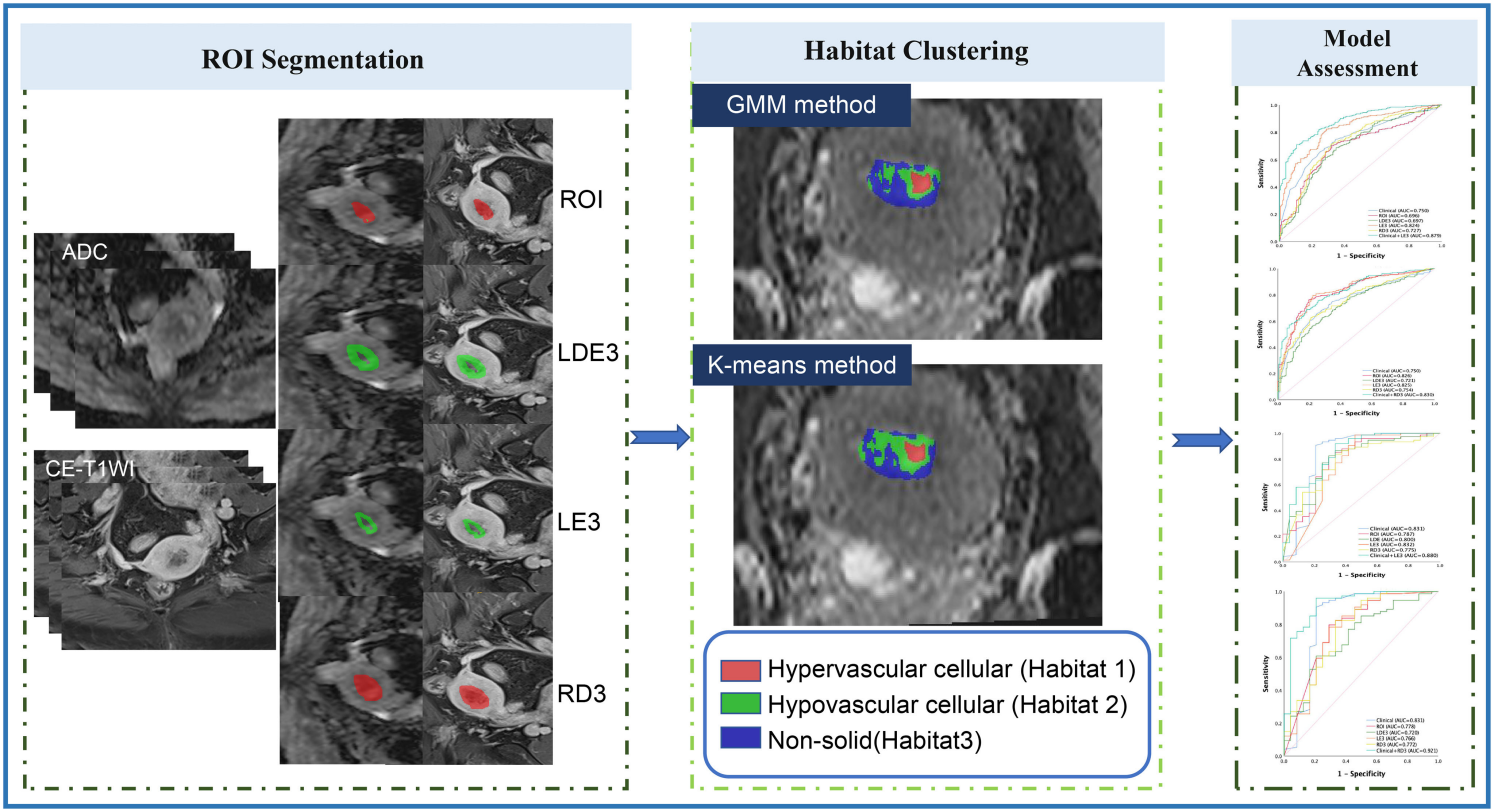
Workflow of ROI segmentation, habitat clustering (GMM/K-means), and diagnostic model evaluation.

Two radiologists, with 5 and 12 years of experience, respectively, manually delineated the regions of interest (ROIs) slice-by-slice along the tumor boundary on the ADC maps, excluding areas of effusion, hemorrhage, and normal myometrium. For tumor boundaries that are poorly defined on the ADC maps, axial T2WI was consulted. The final volume of interest (VOI) was established based on the consensus of the radiologists. Using Python (version 3.11.4, https://www.python.org/), the following peritumoral VOIs were automatically generated: 3-mm dilated and eroded peritumoral loops (LDE3), 3-mm eroded peritumoral loops (LE3), and intratumoral regions combined with 3-mm dilated peritumoral loops (RD3), and named LDE3, LE3, and RD3, respectively (**Figure 2****).** Lesions whose VOI boundaries extended beyond the uterine serosa were excluded.

Initially, CE-T1WI and ADC maps from each patient were registered and resampled to a uniform voxel size. Subsequently, at the individual level, CE-T1WI and ADC maps were used as data inputs for cluster analysis, respectively. The CE-T1WI served as the reference image, and the ADC maps as the moving image. All voxels within the ROI were aggregated into n × 2 global matrices, representing the total n pixels obtained from each sequence. Afterwards, cohort level clustering involved aggregating the voxel matrices of all patients, which were subsequently clustered by the K-means and Gaussian Mixture Model (GMM) method, into a comprehensive global matrix. Consistency clustering was then performed using Python (version 3.11.4, https://www.python.org/) to identify voxel-wise consistency patterns at the cohort level. Using the K-means and GMM algorithms from the scikit-learn package (https://scikit-learn.org/stable/index.html), the voxels within the VOI were encoded with an optimized number of clusters to prevent overfitting and ensure interpretability, settling on three clusters. The original ROI, LDE3, LE3, and RD3 voxels were encoded into distinct habitats based on these results. Finally, habitat parameters, including the volume, percentage, and the mean signal intensity of the habitat, were extracted utilizing the open-source software Feature Explorer (FAE, version 0.5.8).

The clustering of ADC maps and CE-T1WI was defined as functional habitat imaging. Functional habitats were categorized into three groups: Habitat 1 (Low ADC, High CE−T1): Hypervascular cellular (active) region; Habitat 2 (Low ADC, Low CE−T1): Hypovascular cellular (hypoxic/fibrotic) region; Habitat 3 (High ADC, Low CE−T1): Non−viable (necrotic/edematous) region. **Figure 2** shows the flowchart of the habitat imaging process.

### 2.4 Model construction

Univariate and multivariate logistic regression analyses were performed to identify clinical and habitat parameters that could serve as independent predictors. A comparison of clinical and functional habitat parameters was performed between the stage IA EC group and the benign group in the training group. Only the parameters with significant differences were included in univariate logistic regression (*P* < 0.05) and multivariate logistic regression (*P* < 0.05) analyses to screen for clinical and functional habitat independent predictors.

Clinical models were constructed based on clinically independent predictors. Habitat models were constructed based on habitat parameters, encompassing intra- and peri-tumor habitat models for the original ROI, LDE3, LE3, and RD3. To determine the optimal habitat models for ensemble construction, we systematically evaluated the diagnostic performance of all subregion models (including ROI, LDE3, LE3, and RD3) generated by both GMM and K-means. The optimal habitat model was selected based on two primary criteria: (1) robustness, defined by the consistency of AUC values across the training and three external validation cohorts with minimal overfitting; and (2) complementarity, specifically the potential to improve predictive accuracy when combined with clinical factors. Combined models were constructed by integrating the clinical independent predictors and the optimal habitat models.

### 2.5 Statistical analyses

Statistical analysis was conducted with SPSS 26.0 (IBM, New York, USA), and R software 4.1.2 (https://www.r-project.org/). Continuous variables were summarized as medians with interquartile ranges (IQRs) after confirming non-normality with the Shapiro-Wilk test. Categorical variables were expressed as numbers and percentages. Categorical variables were analyzed using the Pearson’s chi-square test or Fisher’s exact test, whereas continuous variables were evaluated using, Mann–Whitney U test, or Kruskal-Wallis test. Univariate and multivariate logistic regression analyses were used to filter the clinical and habitat independent predictors. The diagnostic performance of different models was evaluated and compared using receiver operating characteristic curves (ROC), the area under the curve (AUC), sensitivity, specificity, accuracy, positive predictive value (PPV), and negative predictive value (NPV). Odds ratios (ORs) with 95% confidence intervals (CIs) were calculated. Decision Curve Analysis (DCA) was performed on the average of external validation group to compare the clinical net benefit of the optimal model derived from each of the GMM and K-means clustering methods. Statistical significance was set at *P* < 0.05.

## 3 Results

### 3.1 Clinical characteristics

The clinical characteristics of patients in the training and the validation groups are summarized in **Table 1**. Logistic regression analysis of the clinical characteristics in the training group is shown in **Table 2**. Univariate logistic regression showed significant differences in age, vaginal bleeding and menopausal status (*P* < 0.05). Multivariate logistic regression analysis demonstrated that age and vaginal bleeding (OR = 1.042, 95%CI= 1.015- 1.069; OR = 4.509, 95%CI= 2.911- 6.984, *P* < 0.01) were independent clinical predictors of stage IA EC. The comparison of clinical characteristics between Stage IA endometrial cancer and benign lesions for each center is presented in **Supplementary Tables 2**–**S5**, respectively.

**Table 1:** Clinical characteristics between the training and external validation groups.

Clinical characteristics	Training group	External validation group B	External validation group C	External validation group D
	(n=476)	(n=98)	(n=122)	(n=91)
Age (years)	51.00 (46.00, 56.00)	55.50 (49.00, 63.75)	50.00 (43.00, 54.00)	51.00 (46.00, 59.00)
BMI (kg/m2)	24.44 (22.27, 26.94)	24.20 (21.96, 26.62)	24.61 (22.65, 27.21)	24.03 (21.85, 28.40)
CA199 (U/ml)	11.27 (4.87, 24.88)	10.05 (2.58, 21.85)	11.87 (6.41, 19.77)	13.05 (8.52, 26.60)
CA125 (U/ml)	19.00 (14.00, 30.00)	20.45 (12.93, 40.18)	18.94 (13.43, 25.97)	20.25 (13.75, 38.25)
Tumor grade				
Benign	152 (31.9%)	24 (24.5%)	44 (36.1%)	40 (44.0%)
Malignancy	324 (68.1%)	74 (75.5%)	78 (63.9%)	51 (56.0%)
Vaginal bleeding				
Yes	280 (58.8%)	78 (79.6%)	85 (69.7%)	62 (68.1%)
No	196 (41.2%)	20 (20.4%)	37 (30.3%)	29 (31.9%)
Menopausal				
Yes	214 (45.2%)	64 (65.3%)	56 (45.9%)	43 (47.3%)
No	259 (54.8%)	34 (34.7%)	66 (54.1%)	48 (52.7%)
Metabolic syndrome				
Yes	146 (30.7%)	26 (26.5%)	41 (33.6%)	22 (24.2%)
No	330 (69.3%)	72 (73.5%)	81 (66.4%)	69 (75.8%)

BMI, body mass index; CA125, cancer antigen125; CA199, cancer antigen199.

**Table 2:** Univariate and multivariate LR analysis of clinical characteristics between the stage IA EC and benign endometrial lesions patients in the training group.

Clinical characteristics	Univariate LR			Multivariate LR		
	OR	95%CI	*P*	OR	95%CI	*P*
Age (years)	1.070	1.044-1.097	<0.001*	1.042	1.015- 1.069	0.002*
BMI (kg/m2)	0.960	0.914- 1.008	0.103			
CA199 (U/ml)	1.007	0.999- 1.014	0.079			
CA125 (U/ml)	0.999	0.996-1.002	0.606			
Vaginal bleeding	5.221	3.442- 7.920	<0.001*	4.509	2.911- 6.984	<0.001*
Menopausal	2.799	1.847- 4.243	<0.001*			
Metabolic syndrome	1.238	0.809- 1.894	0.325			

LR, logistic regression; OR, odds ratio; CI, confidence interval; Univariate LR, *P* value of < 0.05; Multivariate LR, *P* value of < 0.05; was considered to indicate significant difference (*).

### 3.2 Habitat parameter selection

The univariate and multivariate logistic regression analyses for habitat parameters are shown in **Supplementary Tables 6**, **S7**.

### 3.3 Performance evaluation of different models

**Figure 3:**
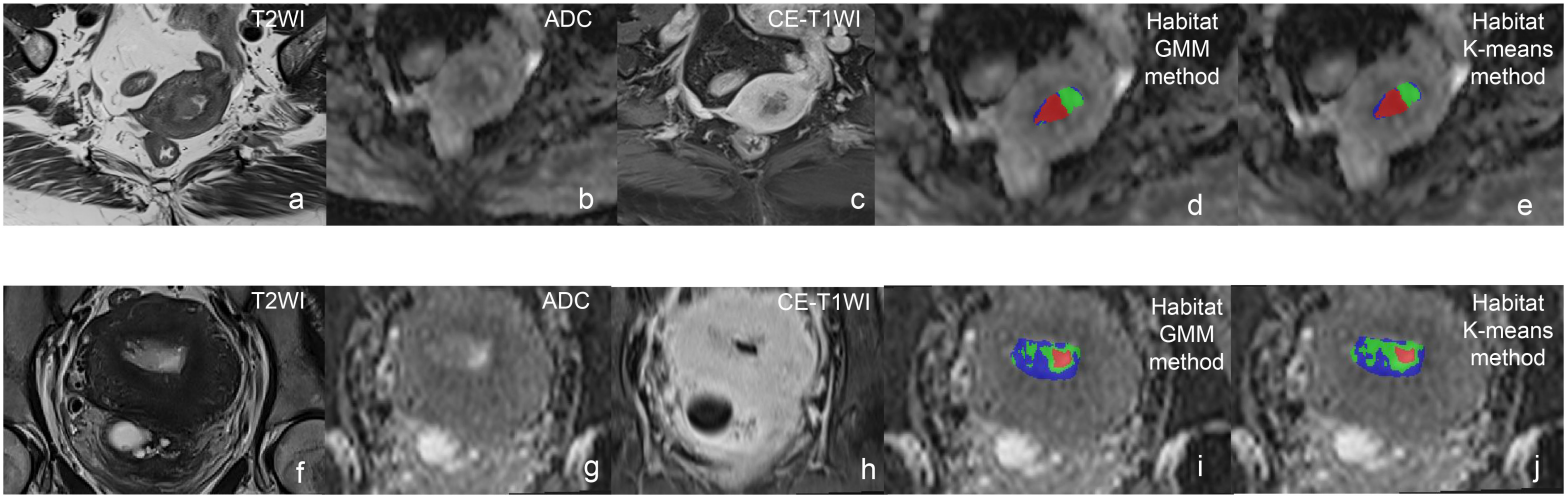
MRI habitat imaging. Case 1: A 63-year-old woman with endometrial cancer **(a–e)**. The red areas represent hypervascular cellular tissue. The green areas represent hypovascular cellular tissue. The blue areas represent non-solid components. Case 2: A 49-year-old woman with endometrial hyperplasia **(f–j)**.

The habitat images clustered using the GMM and K-means based on functional habitat model at the cohort-level clustering are illustrated in **Figure 3**. The diagnostic performances of habitat models based on GMM and K-means are summarized in **Tables 3**, **4**. Overall, the habitat-based models exhibited superior predictive performance compared to the standalone clinical model. This advantage was particularly evident in the more challenging external validation group D, where the AUC of the clinical model was 0.644, while the AUC of multiple habitat models all reached above 0.722, demonstrating better discriminative ability. Although the overall AUC value of the model was at a medium level in multi-center external validation, the habitat model still demonstrated superior stability and generalization ability compared to the clinical model.

**Table 3:** The performance of the clinical and GMM habitat models.

	Models	AUC (95%CI)	Accuracy (95%CI)	Sensitivity (95%CI)	Specificity (95%CI)	PPV (95%CI)	NPV (95%CI)
Training group	Clinical	0.750 (0.705-0.795)	0.687 (0.643-0.728)	0.657 (0.606-0.709)	0.750 (0.681-0.819)	0.849 (0.804-0.893)	0.507 (0.441-0.572)
	GMM_ROI	0.696 (0.647-0.746)	0.693 (0.650-0.734)	0.704 (0.654-0.753)	0.671 (0.596-0.746)	0.820 (0.775-0.865)	0.515 (0.446-0.585)
	GMM_LDE3	0.697 (0.646-0.748)	0.672 (0.628-0.714)	0.682 (0.631-0.733)	0.651 (0.576-0.727)	0.807 (0.760-0.853)	0.490 (0.421-0.559)
	GMM_LE3	0.824 (0.786-0.863)	0.786 (0.746-0.822)	0.821 (0.779-0.863)	0.711 (0.638-0.783)	0.858 (0.819-0.897)	0.651 (0.578-0.723)
	GMM_RD3	0.727 (0.677-0.776)	0.700 (0.656-0.740)	0.707 (0.657-0.756)	0.684 (0.610-0.758)	0.827 (0.782-0.871)	0.523 (0.453-0.592)
	Clinical+ GMM_LE3	0.879 (0.848-0.909)	0.768 (0.728-0.806)	0.709 (0.659-0.759)	0.895 (0.846-0.944)	0.935 (0.904-0.966)	0.591 (0.528-0.655)
External validation group B	Clinical	0.831 (0.702-0.960)	0.878 (0.796-0.935)	0.905 (0.839-0.972)	0.792 (0.629-0.954)	0.931 (0.872-0.989)	0.731 (0.560-0.901)
	GMM_ROI	0.787 (0.671-0.904)	0.786 (0.691-0.862)	0.811 (0.722-0.900)	0.708 (0.526-0.890)	0.896 (0.822-0.969)	0.548 (0.373-0.724)
	GMM_LDE3	0.800 (0.697-0.903)	0.755 (0.658-0.836)	0.767 (0.670-0.864)	0.720 (0.544-0.896)	0.889 (0.811-0.966)	0.514 (0.349-0.680)
	GMM_LE3	0.832 (0.736-0.928)	0.786 (0.691-0.862)	0.778 (0.682-0.874)	0.808 (0.656-0.959)	0.918 (0.849-0.987)	0.568 (0.408-0.727)
	GMM_RD3	0.775 (0.665-0.886)	0.796 (0.703-0.871)	0.838 (0.754-0.922)	0.667 (0.478-0.855)	0.886 (0.811-0.960)	0.571 (0.388-0.755)
	Clinical+ GMM_LE3	0.880 (0.811-0.949)	0.796 (0.703-0.871)	0.778 (0.682-0.874)	0.846 (0.707-0.985)	0.933 (0.870-0.996)	0.579 (0.422-0.736)
External validation group C	Clinical	0.771 (0.683-0.858)	0.754 (0.668-0.828)	0.808 (0.720-0.895)	0.659 (0.519-0.799)	0.808 (0.720-0.895)	0.659 (0.519-0.799)
	GMM_ROI	0.749 (0.655-0.843)	0.762 (0.677-0.835)	0.769 (0.676-0.863)	0.750 (0.622-0.878)	0.845 (0.761-0.929)	0.647 (0.516-0.778)
	GMM_LDE3	0.792 (0.714-0.871)	0.779 (0.695-0.849)	0.731 (0.632-0.829)	0.864 (0.762-0.965)	0.905 (0.832-0.977)	0.644 (0.522-0.766)
	GMM_LE3	0.828 (0.750-0.906)	0.820 (0.740-0.883)	0.821 (0.735-0.906)	0.818 (0.704-0.932)	0.889 (0.816-0.961)	0.720 (0.596-0.844)
	GMM_RD3	0.796 (0.720-0.872)	0.779 (0.695-0.849)	0.744 (0.647-0.840)	0.841 (0.733-0.949)	0.892 (0.817-0.968)	0.649 (0.525-0.773)
	Clinical+ GMM_LE3	0.897 (0.836-0.958)	0.861 (0.786-0.917)	0.885 (0.814-0.956)	0.818 (0.704-0.932)	0.896 (0.828-0.964)	0.800 (0.683-0.917)
External validation group D	Clinical	0.644 (0.526-0.761)	0.670 (0.564-0.765)	0.765 (0.648-0.881)	0.550 (0.396-0.704)	0.684 (0.564-0.805)	0.647 (0.486-0.808)
	GMM_ROI	0.803 (0.710-0.896)	0.791 (0.693-0.869)	0.725 (0.603-0.848)	0.875 (0.773-0.977)	0.881 (0.783-0.979)	0.714 (0.588-0.841)
	GMM_LDE3	0.722 (0.616-0.827)	0.659 (0.553-0.755)	0.510 (0.373-0.647)	0.850 (0.739-0.961)	0.812 (0.677-0.948)	0.576 (0.450-0.702)
	GMM_LE3	0.756 (0.654-0.858)	0.733 (0.630-0.821)	0.788 (0.677-0.899)	0.658 (0.507-0.809)	0.759 (0.645-0.873)	0.694 (0.544-0.845)
	GMM_RD3	0.746 (0.644-0.848)	0.681 (0.575-0.775)	0.529 (0.392-0.666)	0.875 (0.773-0.977)	0.844 (0.718-0.970)	0.593 (0.468-0.719)
	Clinical+ GMM_LE3	0.813 (0.721-0.905)	0.800 (0.702-0.877)	0.788 (0.677-0.899)	0.816 (0.693-0.939)	0.854 (0.754-0.954)	0.738 (0.605-0.871)
The average of external validation group	Clinical	0.714 (0.66–0.766)	0.762 (0.72–0.804)	0.872 (0.828–0.916)	0.556 (0.472–0.644)	0.789 (0.755–0.823)	0.702 (0.62–0.783)
	GMM_ROI	0.635 (0.571-0.699)	0.611 (0.554-0.665)	0.567 (0.495-0.636)	0.694 (0.598-0.779)	0.777 (0.701-0.841)	0.460 (0.382-0.540)
	GMM_LDE3	0.597 (0.532-0.662)	0.669 (0.613-0.721)	0.861 (0.806-0.906)	0.312 (0.227-0.408)	0.699 (0.638-0.755)	0.548 (0.417-0.675)
	GMM_LE3	0.603 (0.539-0.668)	0.535 (0.478-0.592)	0.406 (0.338-0.477)	0.778 (0.688-0.852)	0.774 (0.682-0.849)	0.412 (0.344-0.483)
	GMM_RD3	0.616 (0.553-0.679)	0.537 (0.480-0.593)	0.365 (0.298-0.435)	0.861 (0.781-0.920)	0.831 (0.737-0.902)	0.419 (0.353-0.487)
	Clinical+ GMM_LE3	0.762 (0.704-0.821)	0.758 (0.706-0.805)	0.832 (0.773-0.881)	0.620 (0.522-0.712)	0.804 (0.743-0.855)	0.663 (0.562-0.754)

GMM, Gaussian Mixture Model; AUC, area under the curve; PPV, positive predictive value; NPV, negative predictive value; LDE3, 3-mm dilated and eroded peritumoral loops; LE3, 3-mm eroded peritumoral loops; and RD3, intratumoral regions combined with 3-mm dilated peritumoral loops.

**Table 4:** The performance of the clinical and K-means habitat models.

	Models	AUC (95%CI)	Accuracy (95%CI)	Sensitivity (95%CI)	Specificity (95%CI)	PPV (95%CI)	NPV (95%CI)
Training group	Clinical	0.750 (0.705-0.795)	0.687 (0.643-0.728)	0.657 (0.606-0.709)	0.750 (0.681-0.819)	0.849 (0.804-0.893)	0.507 (0.441-0.572)
	K-means_ROI	0.826 (0.787-0.865)	0.773 (0.733-0.810)	0.759 (0.713-0.806)	0.803 (0.739-0.866)	0.891 (0.855-0.928)	0.610 (0.542-0.678)
	K-means_LDE3	0.721 (0.673-0.768)	0.630 (0.585-0.674)	0.568 (0.514-0.622)	0.763 (0.696-0.831)	0.836 (0.787-0.885)	0.453 (0.392-0.514)
	K-means_LE3	0.825 (0.786-0.864)	0.790 (0.751-0.826)	0.796 (0.752-0.840)	0.776 (0.710-0.843)	0.884 (0.847-0.920)	0.641 (0.572-0.711)
	K-means_RD3	0.754 (0.710-0.799)	0.679 (0.635-0.720)	0.627 (0.574-0.679)	0.789 (0.725-0.854)	0.864 (0.820-0.908)	0.498 (0.435-0.561)
	Clinical+ K-means_RD3	0.830 (0.793-0.867)	0.729 (0.687-0.768)	0.688 (0.638-0.739)	0.816 (0.754-0.877)	0.888 (0.849-0.927)	0.551 (0.486-0.616)
External validation group B	Clinical	0.831 (0.702-0.960)	0.878 (0.796-0.935)	0.905 (0.839-0.972)	0.792 (0.629-0.954)	0.931 (0.872-0.989)	0.731 (0.560-0.901)
	K-means_ROI	0.778 (0.662-0.893)	0.776 (0.680-0.854)	0.797 (0.706-0.889)	0.708 (0.526-0.890)	0.894 (0.820-0.968)	0.531 (0.358-0.704)
	K-means_LDE3	0.720 (0.599-0.840)	0.653 (0.550-0.746)	0.608 (0.497-0.719)	0.792 (0.629-0.954)	0.900 (0.817-0.983)	0.396 (0.257-0.534)
	K-means_LE3	0.766 (0.640-0.892)	0.765 (0.669-0.845)	0.784 (0.690-0.878)	0.708 (0.526-0.890)	0.892 (0.817-0.968)	0.515 (0.345-0.686)
	K-means_RD3	0.772 (0.652-0.892)	0.786 (0.691-0.862)	0.824 (0.738-0.911)	0.667 (0.478-0.855)	0.884 (0.809-0.960)	0.552 (0.371-0.733)
	Clinical+ K-means_RD3	0.921 (0.851-0.991)	0.918 (0.845-0.964)	0.959 (0.915-1.000)	0.792 (0.629-0.954)	0.934 (0.878-0.990)	0.864 (0.720-1.000)
External validation group C	Clinical	0.771 (0.683-0.858)	0.754 (0.668-0.828)	0.808 (0.720-0.895)	0.659 (0.519-0.799)	0.808 (0.720-0.895)	0.659 (0.519-0.799)
	K-means_ROI	0.784 (0.690-0.877)	0.820 (0.740-0.883)	0.859 (0.782-0.936)	0.750 (0.622-0.878)	0.859 (0.782-0.936)	0.750 (0.622-0.878)
	K-means_LDE3	0.813 (0.725-0.901)	0.828 (0.749-0.890)	0.846 (0.766-0.926)	0.795 (0.676-0.915)	0.880 (0.806-0.954)	0.745 (0.620-0.869)
	K-means_LE3	0.772 (0.676-0.868)	0.820 (0.740-0.883)	0.859 (0.782-0.936)	0.750 (0.622-0.878)	0.859 (0.782-0.936)	0.750 (0.622-0.878)
	K-means_RD3	0.829 (0.749-0.909)	0.820 (0.740-0.883)	0.846 (0.766-0.926)	0.773 (0.649-0.897)	0.868 (0.792-0.944)	0.739 (0.612-0.866)
	Clinical+ K-means_RD3	0.907 (0.852-0.962)	0.820 (0.740-0.883)	0.782 (0.690-0.874)	0.886 (0.793-0.980)	0.924 (0.860-0.988)	0.696 (0.576-0.817)
External validation group D	Clinical	0.644 (0.526-0.761)	0.670 (0.564-0.765)	0.765 (0.648-0.881)	0.550 (0.396-0.704)	0.684 (0.564-0.805)	0.647 (0.486-0.808)
	K-means_ROI	0.794 (0.702-0.887)	0.747 (0.645-0.833)	0.686 (0.559-0.814)	0.825 (0.707-0.943)	0.833 (0.721-0.946)	0.673 (0.542-0.805)
	K-means_LDE3	0.796 (0.701-0.890)	0.769 (0.669-0.851)	0.843 (0.743-0.943)	0.675 (0.530-0.820)	0.768 (0.657-0.878)	0.771 (0.632-0.911)
	K-means_LE3	0.805 (0.715-0.894)	0.758 (0.657-0.842)	0.667 (0.537-0.796)	0.875 (0.773-0.977)	0.872 (0.767-0.977)	0.673 (0.546-0.801)
	K-means_RD3	0.818 (0.728-0.908)	0.769 (0.669-0.851)	0.686 (0.559-0.814)	0.875 (0.773-0.977)	0.875 (0.773-0.977)	0.686 (0.559-0.814)
	Clinical+ K-means_RD3	0.850 (0.763-0.937)	0.846 (0.755-0.913)	0.882 (0.794-0.971)	0.800 (0.676-0.924)	0.849 (0.753-0.945)	0.842 (0.726-0.958)
The average of external validation group	Clinical	0.714 (0.66–0.766)	0.762 (0.72–0.804)	0.872 (0.828–0.916)	0.556 (0.472–0.644)	0.789 (0.755–0.823)	0.702 (0.62–0.783)
	K-means_ROI	0.552 (0.470-0.635)	0.609 (0.541-0.674)	0.625 (0.543-0.702)	0.574 (0.448-0.693)	0.766 (0.682-0.837)	0.406 (0.307-0.511)
	K-means_LDE3	0.716 (0.653-0.779)	0.717 (0.663-0.766)	0.759 (0.694-0.816)	0.639 (0.541-0.729)	0.798 (0.734-0.852)	0.585 (0.490-0.675)
	K-means_LE3	0.598 (0.530-0.665)	0.698 (0.643-0.748)	0.906 (0.858-0.943)	0.306 (0.221-0.402)	0.710 (0.651-0.765)	0.635 (0.490-0.764)
	K-means_RD3	0.740 (0.680-0.799)	0.749 (0.697-0.796)	0.798 (0.736-0.851)	0.657 (0.560-0.746)	0.814 (0.753-0.866)	0.634 (0.538-0.723)
	Clinical+ K-means_RD3	0.826 (0.776-0.876)	0.768 (0.718-0.814)	0.744 (0.678-0.802)	0.815 (0.729-0.883)	0.883 (0.825-0.927)	0.629 (0.543-0.709)

AUC, area under the curve; PPV, positive predictive value; NPV, negative predictive value; LDE3, 3-mm dilated and eroded peritumoral loops; LE3, 3-mm eroded peritumoral loops; and RD3, intratumoral regions combined with 3-mm dilated peritumoral loops.

Regarding the comparison between whole-tumor and peritumoral regions, peritumoral habitat features demonstrated clear superiority in terms of generalizability and robustness across multicenter datasets compared to whole-tumor ROI-based models. Models incorporating peritumoral subregions, particularly LE3 and RD3, consistently achieved higher or more stable AUC values when moving from the training group to external validation cohorts. Among all habitat components, K-means_RD3 emerged as the optimal feature for clinical integration. Although its standalone performance in the training group was only moderate (AUC 0.754) relative to the stronger K-means_ROI model (AUC 0.826), it exhibited substantially better generalizability in the external validation cohorts (AUC range: 0.772–0.829), attaining the highest external average AUC of 0.740 (95% CI: 0.680–0.799) among all single habitat models. In contrast, the GMM_ROI model showed marked instability, underperforming the clinical model in the training group (AUC 0.696 vs. 0.750) despite a temporary spike in external validation group D (AUC 0.803). Similarly, GMM_LE3, despite a strong training AUC of 0.824, experienced a sharp decline to an external average of 0.603 (95% CI: 0.539–0.668), highlighting its limited robustness.

**Figure 4:**
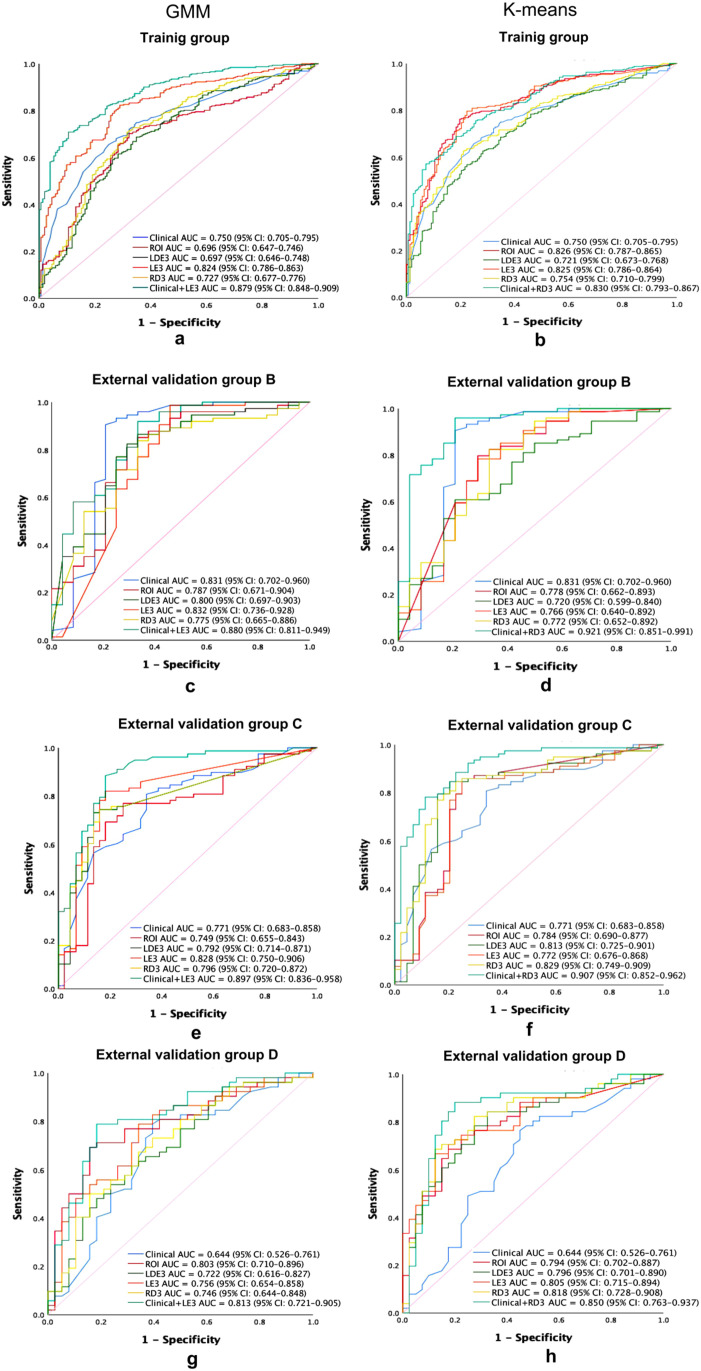
Receiver operating characteristic (ROC) curves of different models. The ROC curves for the clinical and habitat models based on GMM are plotted for the training group **(a)**, external validation group B **(c)**, external validation group C **(e)**, and external validation group D **(g)**. Similarly, the ROC curves for the clinical and habitat models based on K-means algorithm are presented for the training group **(b)**, external validation group B **(d)**, external validation group C **(f)**, and external validation group D **(h)**. The Clinical + K-means_RD3 model achieved the highest external average AUC of 0.826 (95% CI: 0.776–0.876) and reached a peak AUC of 0.921 (95% CI: 0.851–0.991) in external validation group (B) Peritumoral habitat features (LE3 and RD3) provided better generalizability than intratumoral ROI, and K-means clustering outperformed GMM in external validation.

**Figure 5:**
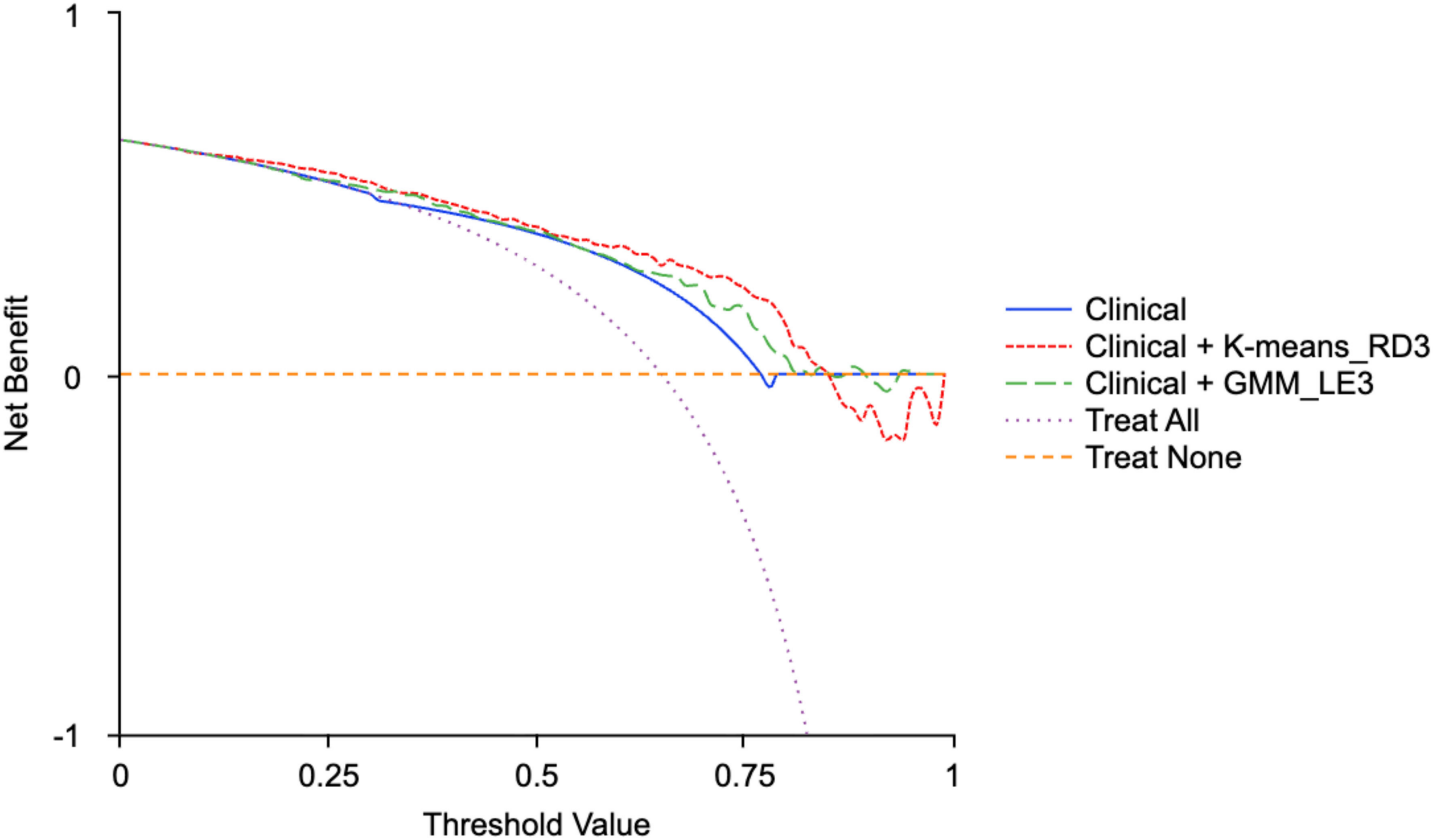
Decision curve analysis (DCA) of the three prediction models. The net benefit curves for these prognostic models are shown. The x-axis indicates the threshold probability, and the y-axis indicates the net benefit. The red dashed curve represents the Clinical + K-means_RD3 combined model, which demonstrates the highest net benefit across a wide range of clinically relevant threshold probabilities compared to the Clinical model and the GMM-based combined model.

When comparing the two clustering algorithms, GMM_LE3 and K-means_RD3 displayed markedly different performance patterns. In the training group, GMM_LE3 outperformed K-means_RD3 (0.824 vs. 0.754). However, this advantage disappeared in external validation. K-means_RD3 maintained more stable performance across the three external cohorts (AUCs of 0.772, 0.829, and 0.818), resulting in a higher external average AUC than GMM_LE3. After integration with clinical variables, the clinical + K-means_RD3 model achieved the highest external average AUC of 0.826 (95% CI: 0.776–0.876), clearly outperforming the clinical + GMM_LE3 model (0.762, 95% CI: 0.704–0.821). The ROC curves for both intratumoral and peritumoral habitat imaging models are shown in **Figure 4**. As shown in **Figure 5**, decision curve analysis revealed that the Clinical + K-means_RD3 model provided the greatest net benefit across a wide range of clinically relevant threshold probabilities compared with the standalone clinical model and the Clinical + GMM_LE3 model in the average of external validation group.

## 4 Discussion

In this study, we developed intratumoral and peritumoral habitat models based on multiparametric MRI to distinguish stage IA EC from benign endometrial lesions. Our findings indicate that all habitat models significantly outperform clinical models in diagnostic efficacy, with the LE3 and RD3 models exhibiting the most robust performance. While both GMM and K-means clustering methods were employed, the K-means-based models demonstrated superior overall generalizability in external validation. The combined Clinical + K-means_RD3 model achieved the highest external average AUC.

The necessity for such advanced diagnostic tools is underscored by the limitations of conventional clinical assessments. Although irregular vaginal bleeding is a typical early symptom of endometrial cancer and serves as a clinically independent predictor along with age, benign diseases (such as endometrial hyperplasia and polyps) also commonly present with abnormal bleeding in postmenopausal women, resulting in a high degree of overlap in clinical features (13–15). This symptom overlap is the primary reason for the relatively low AUC observed in both the training and validation groups of the clinical model. The integration of advanced imaging models into gynecological diagnostics aligns with a growing trend toward precision oncology. Similar to a recent study that successfully employed radiomics and machine learning to differentiate uterine sarcomas (16), our work underscores the significant value of non-invasive habitat imaging.

Through this technique, tumors are partitioned into multiple subregions via voxel clustering, which enables the identification of distinct cellular subpopulations and the inference of tumor heterogeneity (17). This approach allows for the spatial assessment of tumor biology, providing independent information (18, 19). The success of MRI-based habitat analysis in predicting other gynecological malignancies, such as uterine sarcomas (12), prompted its application in our study. Our results confirm its utility: consistent with previous findings (11), these functional habitat models yielded significantly superior predictive performance compared to the clinical model. The Clinical + K-means_RD3 model, in particular, achieved the highest external average AUC (0.907), highlighting the synergistic value of combining clinical predictors with advanced imaging features.

A key contribution of our study is the identification of significant predictive capacity in the regions surrounding the tumor boundary, particularly within the LE3 and RD3 habitats. Our approach was inspired by prior research demonstrating the utility of peritumoral regions in predicting lymph node metastasis and risk stratification in EC patients (20, 21). Specifically, studies by Yan et al. (22) and Ren et al. (23) validated the diagnostic value of a 3-mm peritumoral dimension, providing a strong rationale for our methodological choice to focus on 3-mm inner and outer loops. We hypothesized that a peritumoral loop of 3 mm could also represent information about the tumor area. LE3 region represents the region defined by a 3 mm inward erosion corresponds to the tumor invasive front. Biologically, this interface is characterized by high proliferative activity, neo-angiogenesis, and immune cell infiltration. Unlike the tumor core, which may exhibit heterogeneous necrosis or hypoxia that confounds radiomic features, the peritumoral habitat (LE3) effectively captures these subtle, voxel-level microenvironmental alterations. We find that the GMM-based LE3 exhibited high predictive performance, confirming that assessing this marginal activity is superior to analyzing the whole tumor for distinguishing true malignancy. This predictive superiority of marginal activity is not limited to tumor differentiation; a study by Wang et al. (24) on the assessment of cervical stromal invasion in early-stage EC also indicated that the L3 functional habitat exhibited the highest predictive performance. This collective evidence leads us to speculate that the robustness of LE3 stems from its ability to capture the aggressive biological signatures at the tumor boundary—signatures that potentially indicate microinvasion. Furthermore, RD3 integrates intratumoral and peritumoral information from a 3 mm outward expansion, showing results similar to those of previous studies, which also highlight the significance of the peritumoral region in stage IA EC patients (20, 21). It also further confirms the necessity of extracting peritumoral features from MRI images in stage IA EC patients.

To ensure the reliability of our findings, we further compared two distinct clustering algorithms: GMM and K-means. Our results indicate that although the GMM_LE3 model achieved strong performance on the training group (AUC = 0.824), its generalization ability was limited in multi-center external validation (external average AUC = 0.603). In contrast, although the K-means_RD3 model achieved a slightly lower AUC on the training group (0.754), it demonstrated better stability and generalization across the three external validation groups (AUC range: 0.772–0.829; external average AUC = 0.740). According to the relevant literature (25), GMM is a relatively flexible model that estimates the probability density of data through Gaussian mixture distributions, resulting in hyperellipsoidal cluster boundaries. This flexibility enables it to capture complex distributions in the training group effectively but may also lead to overfitting when applied to heterogeneous multi-center data. In contrast, the K-means algorithm is relatively straightforward and is primarily suitable for spherically separable data structures. Its spherical assumption allows it to focus on stable and distinct core clusters while overlooking subtle or non-universal patterns. The stability of the K-means_RD3 model in external validation suggests that the features extracted by this method possess strong cross-center consistency. Overall, these results indicate that K-means exhibits better generalization performance than GMM in the multi-center setting of this study. We speculate that this may be attributed to the data structure driven by the biological characteristics of the RD3 region, which aligns more closely with the algorithmic assumptions of K-means.

In addition, we performed decision curve analysis (DCA). As shown in **Figure 5**, when evaluated on the average of external validation groups, the Clinical + K-means_RD3 combined model demonstrated the highest net benefit across a wide range of clinically relevant threshold probabilities, consistently outperforming the clinical model alone and the GMM-based combined model. This further confirms the clinical utility of the proposed model. Crucially, as patients with early-stage (Stage IA) EC can often be effectively managed with conservative progestin-based therapy to preserve fertility (26), our model provides a robust tool for preoperative stratification. This diagnostic precision is of immense significance for young women desiring future childbearing, as it supports more personalized and conservative therapeutic strategies.

However, the study has certain limitations. First, as a retrospective study, it is subject to potential risks of selection and recall bias. Larger prospective studies are therefore needed to validate our findings. Second, molecular data was incomplete in our retrospective cohort, preventing the application of the full 2023 FIGO staging system. As a result, some molecularly high-risk patients may have been analyzed as anatomical stage IA. Future validation using cohorts with complete molecular data is required. Third, although the three habitats and their surrounding microenvironments were clustered, point-to-point correspondence between habitat imaging and MR images could not be achieved. In the future, prospective analysis will be required for histopathological and habitat subregion validation. Lastly, this method, manual delineation of ROIs is time-consuming, which limits its feasibility in clinical practice. Consequently, it will be necessary to develop automated segmentation technology based on deep learning in the future.

## 5 Conclusion

Intratumoral and peritumoral habitat imaging based on multiparametric MRI can non-invasively reveal the microstructural characteristics of stage IA EC. Integrating clinical predictors with habitat models showed good diagnostic performance. The peritumoral habitat model outperformed the intratumoral habitat model. This approach may provide more information for preoperative assessment, potentially reducing reliance on invasive biopsies and facilitating individualized surgical planning.

## Data Availability

The original contributions presented in the study are included in the article/**Supplementary Material**. Further inquiries can be directed to the corresponding authors.
