# Supplementary material for: Intratumoral and peritumoral MRI habitat imaging for differentiating stage IA endometrial cancer from benign endometrial lesions: a multicenter study
Source: Front Oncol. 2026 Jul 9;16:1790637. doi: 10.3389/fonc.2026.1790637 (PMC13391255; doi:10.3389/fonc.2026.1790637)
Supplement: Supplementary file 1 [file Table1.docx]

Supplementary Material

Supplementary Table S1. The parameters of all the MR scanners.

| Center | Scanner | Sequence | Repetition  time (ms) | Echo  time (ms) | Field of  view (mm^2^) | Acquisition Matrix (ms) | Slice  thickness (mm) | Slice  gap (mm) |
| --- | --- | --- | --- | --- | --- | --- | --- | --- |
| Training group | Siemens  Aera 1.5 T | T2WI | 3900 | 90 | 320×320 | 512×512 | 3 | 1.5 |
|  |  | DWI  (b=0 and 1000s/mm^2^) | 5600 | 90 | 200×200 | 256×256 | 4 | 1 |
|  |  | CE-T1WI | 3.41 | 1.3 | 240×240 | 320×320 | 2 | 1.5 |
|  | Siemens Prisma 3.0 T | T2WI | 3,200 | 90 | 200×200 | 320×320 | 3 | 3.6 |
|  |  | DWI  (b=0 and 1000s/mm^2^) | 6,300 | 75 | 250×134 | 72×134 | 3 | 3.6 |
|  |  | CE-T1WI | 2.9 | 1.19 | 220×200 | 288×262 | 3 | 0 |
|  | GE Signa  HDXt 3.0T | T2WI | 3500 | 104 | 200×200 | 240×240 | 3 | 1.5 |
|  |  | DWI  (b=0 and 1000s/mm^2^) | 4250 | 70 | 200×200 | 240×240 | 3 | 1 |
|  |  | CE-T1WI | 3.26 | 1.6 | 240×240 | 350×350 | 3 | 1.5 |
| Validation group B | Siemens Aera 1.5T | T2WI | 5080 | 74 | 280×280 | 269×384 | 4 | 0.8 |
|  |  | DWI (b=0 and 1000s/mm^2^) | 4000 | 68 | 439×274 | 76×128 | 4 | 0.8 |
|  |  | CE-T1WI | 3.36 | 2.69 | 240×240 | 320×240 | 4 | 0.8 |
|  | GE Premier 3.0T | T2WI | 6216 | 99.06 | 240×240 | 256×256 | 4 | 0.8 |
|  |  | DWI (b=0 and 1000s/mm^2^) | 2862 | 55.8 | 240x240 | 148×128 | 4 | 0.8 |
|  |  | CE-T1WI | 5.07 | 2.50 | 300×300 | 320×256 | 6 | 1.2 |
| Validation group C | Philips Ingenia 3.0T | T2WI | 1835 | 100 | 200×200 | 332×284 | 3 | 0.3 |
|  |  | DWI  (b=0 and 1000s/mm^2^) | 5271 | 55 | 200×250 | 80×98 | 3 | 0.3 |
|  |  | CE-T1WI | 3.7 | 1.32 | 400×353 | 288×253 | 5 | -2.5 |
|  | GE Pioneer 3.0T | T2WI | 4904 | 85 | 200×200 | 320×256 | 3 | 0.3 |
|  |  | DWI  (b=0 and 1000s/mm^2^) | 3675 | 50 | 180×144 | 110×72 | 3 | 0.3 |
|  |  | CE-T1WI | 3.5 | 1.7 | 400×360 | 340×256 | 5 | -2.5 |
| Validation group D | Siemens Skyra 3.0T | T2WI | 3000 | 87 | 240×240 | 320×320 | 5 | 1 |
|  |  | DWI (b=0 and 1000s/mm^2^) | 5700 | 92 | 240×240 | 320×256 | 4 | 1 |
|  |  | CE-T1WI | 3.3 | 1. 3 | 240×240 | 320×320 | 3 | 0 |
|  | GE Signa HDxt 1.5T | T2WI | 6680 | 130 | 240×240 | 320×224 | 4 | 0.5 |
|  |  | DWI (b=0 and 1000s/mm^2^) | 7000 | 77.5 | 240×240 | 320×256 | 5 | 1 |
|  |  | CE-T1WI | 3.4 | 1.6 | 240×240 | 320×224 | 3 | 0 |

T2WI: T2-weighted imaging; DWI: diffusion-weighted imaging ; CE-T1WI: late contrast-enhanced T1-weighted imaging.

Supplementary Table S2. Comparison of clinical characteristics between benign and malignant lesions in the Training Cohort (n=476).

| Clinical characteristics | Benign | Malignancy | *p* |
| --- | --- | --- | --- |
| N | 152 | 324 |  |
| Age (years) | 48.00 (44.00, 52.00) | 52.00 (48.00, 57.25) | <0.001 |
| BMI (kg/m^2^) | 24.67 (22.91, 27.29) | 24.26 (22.13, 26.76) | 0.125 |
| CA199 (U/ml) | 8.12 (3.95, 17.50) | 13.93 (5.39, 28.97) | <0.001 |
| CA125 (U/ml) | 18.00 (13.00, 29.25) | 20.00 (14.00, 30.00) | 0.142 |
| Vaginal bleeding |  |  | \| <0.001 \| \| --- \| |
| Yes | 49 (32.2) | 232 (71.6) |  |
| No | 103 (67.8) | 92 (28.4) |  |
| Menopausal |  |  | <0.001 |
| Yes | 43 (28.7) | 171 (52.9) |  |
| No | 107 (71.3) | 152 (47.1) |  |
| Metabolic syndrome |  |  | 0.339 |
| Yes | 42 (27.6) | 104 (32.1) | 42 (27.6) |
| No | 110 (72.4) | 220 (67.9) |  |

BMI: body mass index; CA125, cancer antigen125; CA199, cancer antigen199

Supplementary Table S3. Comparison of clinical characteristics between benign and malignant lesions in Validation Cohort A (n=98).

| Clinical characteristics | Benign | Malignancy | *p* |
| --- | --- | --- | --- |
| N | 24 | 74 |  |
| Age (years) | 60.00 (50.00, 66.00) | 55.00 (49.00, 62.50) | 0.284 |
| BMI (kg/m^2^) | 23.20 (21.86, 26.38) | 24.23 (21.96, 26.77) | 0.455 |
| CA199 (U/ml) | 6.96 (2.58, 14.54) | 11.38 (2.57, 28.41) | 0.54 |
| CA125 (U/ml) | 18.19 (15.10, 105.12) | 21.40 (11.62, 37.93) | 0.687 |
| Vaginal bleeding |  |  | <0.001 |
| Yes | 8 (33.3) | 70 (94.6) |  |
| No | 16 (66.7) | 4 (5.4) |  |
| Menopausal |  |  | 1 |
| Yes | 16 (66.7) | 48 (64.9) |  |
| No | 8 (33.3) | 26 (35.1) |  |
| Metabolic syndrome |  |  | 0.598 |
| Yes | 5 (20.8) | 21 (28.4) |  |
| No | 19 (79.2) | 53 (71.6) |  |

BMI: body mass index; CA125, cancer antigen125; CA199, cancer antigen199

Supplementary Table S4. Comparison of clinical characteristics between benign and malignant lesions in Validation Cohort B (n=122).

| Clinical characteristics | Benign | Malignancy | *p* |
| --- | --- | --- | --- |
| N | 44 | 78 |  |
| Age (years) | 46.00 (36.75, 50.25) | 52.00 (45.00, 56.75) | 0.001 |
| BMI (kg/m^2^) | 23.85 (22.56, 25.62) | 24.84 (22.83, 27.83) | 0.157 |
| CA199 (U/ml) | 10.40 (4.10, 15.15) | 12.86 (7.04, 20.05) | 0.146 |
| CA125 (U/ml) | 19.45 (12.57, 28.73) | 18.85 (14.18, 25.59) | 0.9 |
| Vaginal bleeding |  |  | <0.001 |
| Yes | 19 (43.2) | 66 (84.6) |  |
| No | 25 (56.8) | 12 (15.4) |  |
| Menopausal |  |  | 0.188 |
| Yes | 16 (37.2) | 39 (50.0) |  |
| No | 27 (62.8) | 39 (50.0) |  |
| Metabolic syndrome |  |  | 0.003 |
| Yes | 7 (15.9) | 34 (43.6) |  |
| No | 37 (84.1) | 44 (56.4) |  |

BMI: body mass index; CA125, cancer antigen125; CA199, cancer antigen199

Supplementary Table S5. Comparison of clinical characteristics between benign and malignant lesions in Validation Cohort C (n=91).

| Clinical characteristics | Benign | Malignancy | *p* |
| --- | --- | --- | --- |
| N | 40 | 51 |  |
| Age (years) | 49.50 (45.75, 58.00) | 53.00 (47.00, 59.00) | 0.282 |
| BMI (kg/m^2^) | 23.94 (21.74, 28.62) | 24.97 (22.60, 28.06) | 0.71 |
| CA199 (U/ml) | 11.92 (6.98, 16.35) | 16.70 (9.90, 38.55) | 0.009 |
| CA125 (U/ml) | 20.10 (14.10, 30.40) | 22.10 (13.50, 39.00) | 0.834 |
| Vaginal bleeding |  |  | 0.006 |
| Yes | 21 (52.5) | 41 (80.4) |  |
| No | 19 (47.5) | 10 (19.6) |  |
| Menopausal |  |  | 0.526 |
| Yes | 17 (42.5) | 26 (51.0) |  |
| No | 23 (57.5) | 25 (49.0) |  |
| Metabolic syndrome |  |  | 1 |
| Yes | 10 (25.0) | 12 (23.5) |  |
| No | 30 (75.0) | 39 (76.5) |  |

BMI: body mass index; CA125, cancer antigen125; CA199, cancer antigen199

Table S6. Univariate and multivariate analysis of GMM habitat models parameters.

| GMM habitat models | Univariate LR | | | Multivariate LR | | |
| --- | --- | --- | --- | --- | --- | --- |
|  | OR | 95%CI | *P* | OR | 95%CI | *P* |
| **GMM_ROI** |  |  |  |  |  |  |
| mask1_Volume | 1.000 | 1.000-1.000 | <0.001* | 1.000 | 1.000-1.000 | <0.001* |
| ADCR_mask2_Mean | 0.999 | 0.999-1.000 | 0.002* | 0.999 | 0.999-1.000 | <0.001* |
| CER_mask1_Percent | 2.555 | 1.524-4.283 | <0.001* |  |  |  |
| CER_mask2_Percent | 0.334 | 0.192-0.581 | <0.001* |  |  |  |
| ADCR_mask1_Percent | 2.555 | 1.524-4.283 | <0.001* |  |  |  |
| ADCR_mask2_Percent | 0.334 | 0.192-0.581 | <0.001* |  |  |  |
| **GMM_LDE3** |  |  |  |  |  |  |
| mask1_Volume | 1.000 | 1.000-1.000 | 0.509 |  |  |  |
| ADCR-LDE3_mask2_Mean | 0.995 | 0.993-0.997 | <0.001* | 0.995 | 0.993-0.997 | <0.001* |
| mask2_Volume | 1.000 | 1.000-1.000 | 0.021* | 1.000 | 1.000-1.000 | 0.002* |
| **GMM_LE3** |  |  |  |  |  |  |
| CER_mask1_Mean | 1.000 | 1.000-1.000 | <0.001* | 0.997 | 0.994-1.00 | 0.024* |
| ADCR-LE3_mask1_Mean | 1.000 | 1.000-1.000 | <0.001* | 1.004 | 1.00-1.008 | 0.032* |
| mask1_Volume | 1.000 | 1.000-1.000 | 0.054 |  |  |  |
| ADCR-LE3_mask2_Mean | 0.994 | 0.993-0.996 | <0.001* | 0.993 | 0.992-0.995 | <0.001* |
| mask2_Volume | 1.000 | 1.000-1.000 | 0.001* | 1.000 | 1.000-1.000 | <0.001* |
| mask3_Volume | 1.000 | 1.000-1.000 | 0.470 |  |  |  |
| CER_mask1_Percent | 353.251 | 12.773-9769.305 | <0.001* |  |  |  |
| CER_mask2_Percent | 0.824 | 0.371-1.830 | 0.634 |  |  |  |
| ADCR-LE3_mask1_Percent | 353.251 | 12.773-9769.305 | <0.001* |  |  |  |
| ADCR-LE3_mask2_Percent | 0.824 | 0.371-1.830 | 0.634 |  |  |  |
| \| CER_mask3_Mean \| \| --- \| | 1.000 | 1.000-1.000 | 0.015* | 1.002 | 1.001-1.002 | <0.001* |
| **GMM_RD3** |  |  |  |  |  |  |
| ADCR-RD3_mask1_Mean | 0.994 | 0.993-0.996 | <0.001* | 0.994 | 0.993-0.996 | <0.001* |
| mask1_Volume | 1.001 | 1.000-1.001 | 0.005* | 1.001 | 1.000-1.001 | 0.006* |
| mask2_Volume | 1.000 | 1.000-1.000 | 0.834 |  |  |  |

LR: logistic regression; OR: odds ratio; CI: confidence interval. Univariate LR: *P* value of ˂ 0.05, Multivariate LR : *P* value of ˂ 0.05 , was considered to indicate significant difference (*).

Table S7. Univariate and multivariate analysis of K-means habitat models parameters.

| K-means habitat models | Univariate LR | | | Multivariate LR | | |
| --- | --- | --- | --- | --- | --- | --- |
|  | OR | 95%CI | *P* | OR | 95%CI | *P* |
| **K-means_ROI** |  |  |  |  |  |  |
| ADCR_mask1_Mean | 0.997 | 0.997-0.998 | <0.001* |  |  |  |
| CER_mask2_Mean | 1.001 | 1.001-1.001 | 0.001* | 1.001 | 1.001-1.001 | 0.001* |
| ADCR_mask2_Mean | 1.001 | 1.001-1.001 | 0.016* |  |  |  |
| mask3_Volume | 1.001 | 1.001-1.001 | 0.001* |  |  |  |
| CER_mask1_Percent | 0.014 | 0.005-0.036 | <0.001* |  |  |  |
| CER_mask3_Percent | 196.150 | 59.586-645.704 | <0.001* | 246.765 | 72.376-841.34 | <0.001* |
| ADCR_mask1_Percent | 0.014 | 0.005-0.036 | <0.001* |  |  |  |
| ADCR_mask3_Percent | 196.150 | 59.586-645.704 | <0.001* |  |  |  |
| **K-means_LDE3** |  |  |  |  |  |  |
| CER_mask1_Mean | 1.001 | 1.001-1.001 | 0.003* | 1.001 | 1.001-1.002 | <0.001* |
| ADCR-LDE3_mask1_Mean | 1.001 | 1.001-1.001 | 0.031* |  |  |  |
| mask1_Volume | 1.000 | 1.000-1.000 | 0.463 |  |  |  |
| ADCR-LDE3_mask2_Mean | 0.996 | 0.995-0.995 | <0.001* | 0.996 | 0.994-0.997 | <0.001* |
| CER_mask3_Mean | 1.001 | 1.001-1.002 | <0.001* | 0.999 | 0.997-1.00 | 0.037* |
| ADCR-LDE3_mask3_Mean | 1.002 | 1.001-1.002 | <0.001* | 1.003 | 1.001-1.004 | 0.004* |
| mask3_Volume | 1.001 | 1.001-1.001 | 0.012* |  |  |  |
| CER_mask1_Percent | 2.678 | 0.776-9.238 | 0.119 |  |  |  |
| CER_mask2_Percent | 0.249 | 0.078-0.796 | 0.019* |  |  |  |
| CER_mask3_Percent | 7.561 | 0.791-72.249 | 0.079 |  |  |  |
| ADCR-LDE3_mask1_Percent | 2.678 | 0.776-9.238 | 0.119 |  |  |  |
| ADCR-LDE3_mask2_Percent | 0.249 | 0.078-0.796 | 0.019* |  |  |  |
| ADCR-LDE3_mask3_Percent | 7.561 | 0.791-72.249 | 0.079 |  |  |  |
| **K-means_LE3** |  |  |  |  |  |  |
| CER_mask1_Mean | 1.001 | 1.001-1.001 | <0.001* |  |  |  |
| mask1_Volume | 1.001 | 1.001-1.001 | <0.001* | 1.000 | 1.000-1.001 | 0.008* |
| CER_mask3_Mean | 1.001 | 1.001-1.001 | 0.002* | 1.001 | 1.001-1.001 | 0.001* |
| mask3_Volume | 1.000 | 1.000-1.000 | 0.932 |  |  |  |
| CER_mask1_Percent | 351.172 | 91.525-1347.406 | <0.001* | 89.764 | 16.294-494.523 | <0.001* |
| CER_mask3_Percent | 1.376 | 0.403-4.696 | 0.611 |  |  |  |
| ADCR-LE3_mask1_Percent | 351.172 | 91.525-1347.406 | <0.001* |  |  |  |
| ADCR-LE3_mask3_Percent | 1.376 | 0.403-4.696 | 0.611 |  |  |  |
| ADCR-LE3_mask2_Mean | 0.997 | 0.996-0.998 | <0.001* |  |  |  |
| CER_mask2_Percent | 0.012 | 0.005-0.034 | <0.001* |  |  |  |
| ADCR-LE3_mask2_Percent | 0.012 | 0.005-0.034 | <0.001* |  |  |  |
| **K-means_RD3** |  |  |  |  |  |  |
| ADCR-RD3_mask1_Mean | 0.996 | 0.995-0.998 | <0.001* | 0.996 | 0.994-0.997 | <0.001* |
| mask1_Volume | 1.001 | 1.001-1.001 | 0.011* | 1.000 | 1.000-1.000 | 0.002* |
| CER_mask2_Mean | 1.001 | 1.001-1.001 | 0.003* | 1.001 | 1.001-1.002 | <0.001* |
| mask2_Volume | 1.000 | 1.000-1.000 | 0.980 |  |  |  |
| CER_mask3_Mean | 1.001 | 1.001-1.002 | <0.001* | 0.998 | 0.997-0.999 | 0.003* |
| ADCR-RD3_mask3_Mean | 1.002 | 1.001-1.003 | <0.001* | 1.003 | 1.002-1.005 | <0.001* |
| mask3_Volume | 1.001 | 1.001-1.001 | 0.003* |  |  |  |
| CER_mask1_Percent | 0.288 | 0.097-0.855 | 0.025* |  |  |  |
| CER_mask3_Percent | 11.000 | 1.244-97.238 | 0.031* |  |  |  |
| ADCR-RD3_mask1_Percent | 0.288 | 0.097-0.855 | 0.025* |  |  |  |
| ADCR-RD3_mask3_Percent | 11.000 | 1.244-97.238 | 0.031* |  |  |  |

LR: logistic regression; OR: odds ratio; CI: confidence interval. Univariate LR *P* value of ˂ 0.05, Multivariate LR *P* value of ˂ 0.05 was considered to indicate significant difference (*).
